# Overcoming harmonic hurdles: Genuine beta-band rhythms vs. contributions of alpha-band waveform shape

**DOI:** 10.1162/imag_a_00018

**Published:** 2023-09-11

**Authors:** Natalie Schaworonkow

**Affiliations:** Ernst Strüngmann Institute for Neuroscience, Frankfurt am Main, Germany

**Keywords:** neural oscillations, alpha-rhythm, beta-rhythm, harmonics, waveform shape, spectral analysis

## Abstract

Beta-band activity in the human cortex as recorded with noninvasive electrophysiology is of diverse origin. In addition to genuine beta-rhythms, there are numerous nonsinusoidal alpha-band rhythms present in the human brain, which will result in harmonic beta-band peaks. This type of activity has different temporal and response dynamics than genuine beta-rhythms. Here, it is argued that in the analysis of higher-frequency rhythms, the relationship to lower-frequency rhythms needs to be clarified. Only in that way we can arrive at strong, methodologically valid interpretations of potential functional roles and generative mechanisms of neural oscillations.

## Many New Beta Studies are Innovative, But they often use Agnostic Band-Pass Filtering

Neural activity recorded from the human brain shows intricate oscillations in different frequency bands. One of the frequency bands showing prominent spectral peaks for brain activity recorded from the human cortex is the beta-band (16–30 Hz). The functional role of these oscillations has therefore been of long-standing interest, with different proposals put forward (Engel & Fries, 2010; Hari, 1997; Spitzer & Haegens, 2017), but no definitive answer emerging yet. Many recent studies have been looking at human cortical beta-band activity and using innovative and robust methodology to study diverse aspects with renewed attention: the burst-like nature of occurrence and the relationship of the beta bursts to task-related outcomes (Chen et al., 2023; Little et al., 2019; Sherman et al., 2016; Shin et al., 2017; Wessel, 2020; West et al., 2023), pushing the boundaries of mapping the spatial specificity and laminar contributions of beta-activity (Barratt et al., 2018; Bonaiuto et al., 2021); the spatial dissociation from alpha-band activity as well as spatial propagation dynamics (Cao et al., 2022; Stolk et al., 2019; Zich et al., 2023), disentangling several distinct types of beta bursts with potentially different functional roles (Rassi et al., 2023; Szul et al., 2023); using large datasets to examine the beta-rhythm heritability (Espenhahn et al., 2017; Pauls et al., 2023) and the potential of beta-activity as a biomarker for motor function (Rempe et al., 2022); testing the causal effects of different beta states with real-time phase stimulation (Wischnewski et al., 2022); and detailed computational modeling for insights into generative mechanisms (Law et al., 2022; Sherman et al., 2016).

**Fig. 1.**
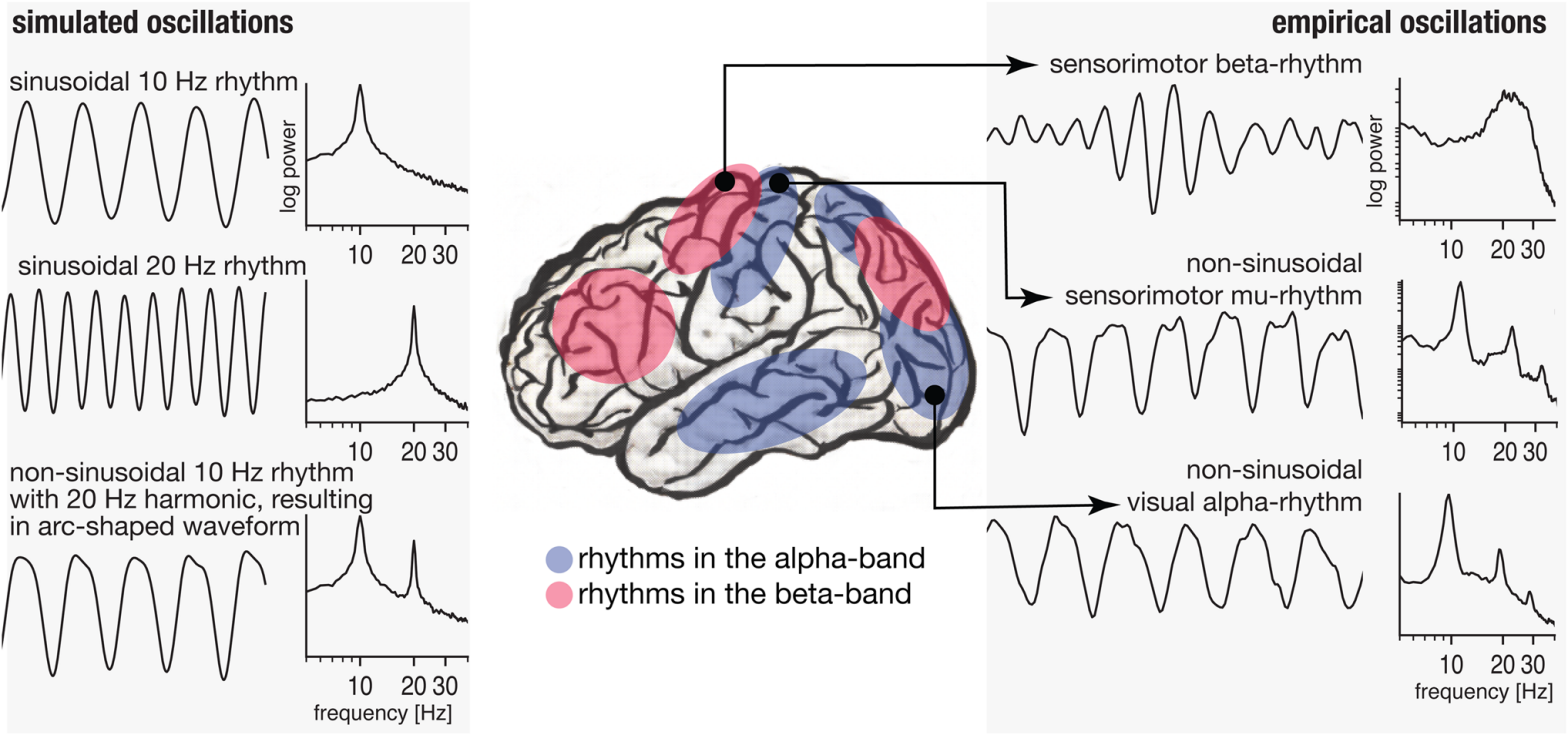
Diversity of cortical alpha- and beta-rhythms present in the human brain. Left: Simulated sinusoidal and nonsinusoidal rhythms with narrowband 10 Hz and 20 Hz contributions. Middle: Locations of prominent rhythm generators of alpha- and beta-rhythms, with some regions showing overlap, locations are adapted from Spitzer and Haegens (2017) and Schaworonkow and Nikulin (2022). Right: Example time series of rhythms in the beta- and alpha-band (high-pass filtered at 1 Hz), with the alpha-band rhythms showing nonsinusoidal waveform shape, resulting in harmonic peaks in the beta-band. Note the broad beta-peak for genuine beta-rhythms compared to the narrow-band harmonic peaks.

However, in the study of beta-rhythms in the human cortex, one crucial aspect has been mostly neglected, that is the existence of prominent lower-frequency alpha-band rhythms and their nonsinusoidal waveform shape (Cole & Voytek, 2017; Schaworonkow & Nikulin, 2019; Stam et al., 1999; van Albada & Robinson, 2013). This results in contributions to activity in the beta-band from the harmonics of these strong alpha-band rhythms. Although genuine beta-activity without an underlying lower-frequency oscillation exists, there is also beta-activity that is a harmonic of alpha-rhythms. For example, this is the case for not only nonsinusoidal rhythms from the sensorimotor cortex but also for occipital rhythms, as illustrated in Figure 1. Harmonic oscillations will generally show very similar types of temporal dynamics as the base rhythm. Usually, at one point in the processing pipeline, a temporal filtering step is employed, with band-pass filtering in a canonical frequency band, e.g., 16–30 Hz in the case of beta-rhythms. This step can collapse across these two sources of origin, making the investigation of the functional roles of different rhythms more challenging, as temporal dynamics and functional modulation of different rhythms will be conflated.

## Sensorimotor Beta-Band Activity has Contributions from Alpha-Band Harmonics

**Fig. 2.**
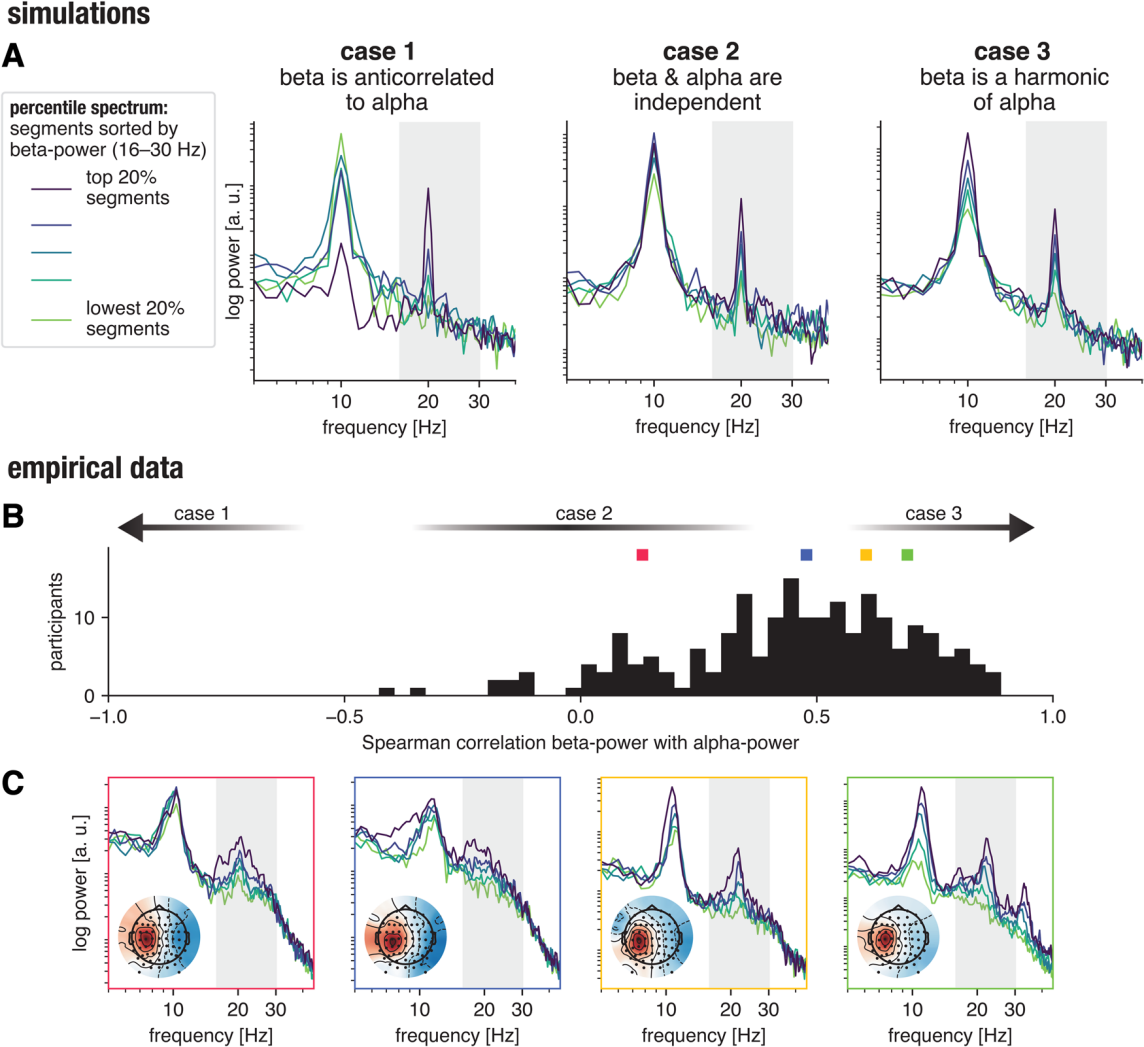
Beta-band activity recorded at a central EEG electrode can be of harmonic origin. (A) Simulated scenarios for the relationship between 1/f-corrected alpha- and beta-power, showing the corresponding percentile spectra, sorted by beta-power (number of groups = 5). The shown cases here are anticorrelation, independence, or positive correlation between alpha- and beta-power. (B) In empirical data (eyes open condition), most participants exhibit a positive correlation between 1/f-corrected alpha- and beta-power, hinting at a harmonic relationship (number of groups = 20). (C) Example participant spectra with correlation values indicated by small squares in (B), with a diversity of possible spectral composition of rhythms visible. Segment length for power spectrum computation was set to 3 seconds. The plots can be reproduced by using the code available under github.com/nschawor/eeg-beta-harmonic.

To demonstrate how band-power varies in other frequency bands, depending on beta-power, we use a percentile spectrum visualization: the spectral power across frequencies was computed for each 2 second segment of the time series. The power in the 16–30 Hz band was then used to sort the segments into a number of groups, and the average spectrum was computed for each beta-power group. This procedure allows us to show the systematic variation of power in other frequency bands during high and low beta-power segments. Figure 2A shows different possible simulated scenarios: case 1—alpha-power and beta-power can be anti-correlated, with high beta-power segments showing no or reduced alpha-power; case 2—alpha-power and beta-power are independent; or case 3—if the beta-rhythm is a harmonic of alpha-rhythm, their power will be positively correlated, with high beta-power resulting in high alpha-power and vice versa. These cases are not mutually exclusive, for example, in the presence of multiple rhythms, but are shown here for demonstration purposes.

To visualize possible scenarios in empirical data, we used a large data set (Babayan et al., 2019) as an example, using left central EEG electrode C3, which is often investigated in the case of sensorimotor processes. We divided the number of segments into 20 groups and computed the Spearman rank correlation between 1/f-corrected alpha- and beta-power (power values are 1/f-corrected by subtracting an 1/f-estimate from the spectrum of each group). As illustrated in Figure 2B, we found the following configuration for a high number of participants: the corresponding alpha-power systematically varies with positive correlation when sorting segments according to beta-power, corresponding to case 3. Figure 2C shows a few example participant percentile spectra sorted for beta-power, sampling the range of variation in alpha-power by varying beta-power. This demonstration is one indication that beta-power has harmonic contributions in the resting state baseline for a number of participants. Of course, the used measure will not capture all possible scenarios, whether there are genuine beta bursts overlapping with harmonic contributions, or concurrent changes in 1/f-dynamics. We do not aim to provide a general measure that is applicable in all scenarios, but merely aim to motivate the reader to check possible relationships in their own data.

## Posterior Alpha-Band Rhythms also show Nonsinusoidal waveform shape for a high number of Participants

**Fig. 3.**
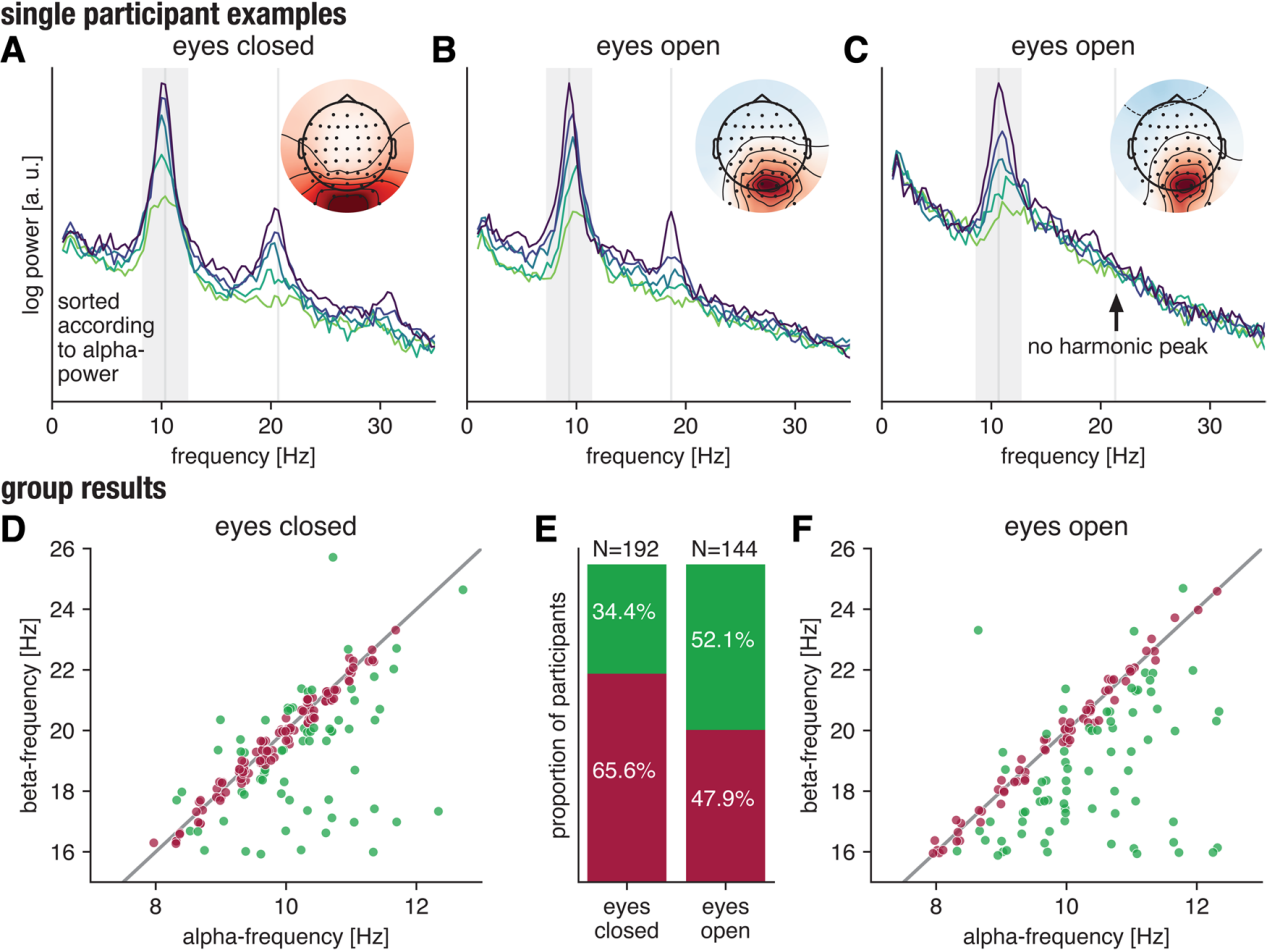
Posterior alpha-band rhythms have harmonic peaks in the beta-band in a high number of participants. (A) Eyes closed condition: percentile spectrum for one participant, with spectra from segments grouped according to their alpha-power. Clearly, a beta-peak emerges for segments with high alpha-power. (B) Eyes open condition: percentile spectrum for one participant. (C) Eyes open condition: This participant is an example where the harmonic peak is absent, even for high alpha-power. (D) Group results—eyes closed condition: individual alpha-peak plotted against individual beta-peak, showing a close alignment. Individual data points are color-coded regarding whether their beta/alpha-peak ratio was close to 2 (violet) or not (green). (E) Percentage of participants with beta/alpha-peak ratio close to 2 (violet) or not close to 2 (green), respectively, for both conditions. (F) Group results—eyes open condition, individual alpha-peak plotted against individual beta-peak (Color coding as in D). Only high alpha-power episodes of rhythms with a SNR > 5 dB were included here, segment length was set to 3 seconds, and a slight jitter was applied on the individual data points for visualization purposes only.

While it is known canonically that the sensorimotor mu-rhythm has a nonsinusoidal arc waveform shape (Gastaut, 1952), here, we also want to highlight that a nonsinusoidal waveform is also present for many posterior alpha-band rhythms. We extracted the rhythm with the highest alpha-band signal-to-noise ratio using a spatial filter for each participant (as in Schaworonkow & Nikulin, 2019), which is a posterior rhythm for the overwhelming majority of the participants and then estimated the alpha- and beta-frequency-peak for segments with high alpha-power (in the top 20%). Figure 3 shows spectral analysis of posterior alpha-rhythms for three example participants, illustrating the presence of harmonic peaks for eyes closed (Fig. 3A) as well as for eyes open (Fig. 3B) states. Not all participants display harmonic peaks, as visible in Figure 3C.

We then examined the relationship between alpha-band activity and beta-band activity for each participant, in terms of correspondence of the peak frequency (Haegens et al., 2014). We determined the peak frequency in alpha- and beta-band in the segments with high alpha-power. Plotting the peak frequencies against each other, a strong preference for harmonic relationship can be seen in Figure 3D and Figure 3F, with beta-frequency being twice the alpha-frequency. We classified the beta-frequency peak as harmonic for 65.6% of participants in the eyes closed case and 47.9% of participants in the eyes open case (Fig. 3E), allowing a deviation of one frequency bin from the linear relationship. This illustrates that harmonic beta-peaks are detectable for many participants, meaning that harmonic beta-activity contributions are a ubiquitous phenomenon in the presence of posterior alpha-rhythms. In the case of the sensor space analysis using channel C3, the same metric yields 26.7% of participants in the eyes closed condition and 32.3% of participants in the eyes open condition, which warrant further investigation. Note that the focus on high alpha-power segments makes this analysis slightly different than in Haegens et al. (2014), as potential harmonic beta-peaks are amplified in high alpha-power segments, as our aim here is to show some contribution of harmonic beta to beta-band activity, but not to claim exclusive harmonic beta-presence.

## Practical Recommendations

Generally, visual inspection of raw or minimally filtered time series remains a crucial technique in ensuring the presence of oscillations in general and not merely reflecting 1/f-activity. To adopt a more systematic approach, time-domain burst analysis can be performed. When using spectral analysis, we recommend visualizing a spectrum or spectrogram across a wide frequency band (not cropping the low frequencies) for better quality control. This can also help to choose appropriate frequency-bands for subsequent band-pass filtering. For general guidelines for analysis of neural oscillations, see Donoghue et al. (2021).

To identify harmonics, it is advisable to check for correspondence in center frequencies, phase locking as well as spatial correspondence of the involved rhythms. For a quick visualization of center frequency correspondence, a percentile spectrum as used above may be useful to inspect power dependencies across frequency bands. This procedure can also identify segments with corresponding high band-power which can then further be investigated via time series analysis. For this demo here, a fixed segment length of 3 seconds was employed. In the resting state data used here, consistent results were observed across participants for a range of segment length values (0.5–4 seconds). However, it’s important to note that outcomes might differ in other datasets, particularly those involving trial-based data. Note that the existence of power dependencies by itself does not constitute harmonics all by itself, but strong narrow-band peaks warrant further careful checks. Recently, identification of harmonics via the instantaneous frequency has been put forward (Fabus et al., 2022). The bispectrum and bicoherence enable us to identify the joint distribution of power in a frequency-resolved manner, with harmonics appearing as localized peaks (Kovach et al., 2018; Shahbazi Avarvand et al., 2018). Another coherence measure to quantify harmonics is cross-frequency lagged coherence, which uses phase consistency across time to disentangle harmonics from genuine rhythms (Fransen et al., 2016). The distinction between harmonic and genuine beta bursts is especially of interest for analyses performed in sensor space, as often done in developmental data (Rayson et al., 2022). If a rhythm is attributed to a harmonic, it may be beneficial to continue the analysis using the underlying base-frequency band instead of the harmonic frequency-band for further analyses, in order to profit from a much higher signal-to-noise ratio of the base frequency rhythm.

## Concluding Remarks

With these demonstrations, we want to highlight the diversity of contributions to activity in the beta-band present in the adult EEG as recorded from the human cortex. The most important aspect we aim to emphasize is that in order to arrive at strong, methodologically valid interpretations of potential functional roles, that in analysis of higher-frequency rhythms the relationship to lower-frequency rhythms needs to be clarified. This is necessary because there are numerous nonsinusoidal rhythms present in the human brain, as quantified using EEG or intracranial recordings (Schaworonkow & Nikulin, 2019; Schaworonkow & Voytek, 2021). For beta-rhythms, this is specifically noteworthy since strong alpha-rhythms often dominate the EEG of healthy adults. The stronger the alpha-band activity is, the stronger the harmonic beta-band activity will be, simply given due to the linearity of the Fourier transform. Nonsinusoidal waveform has a strong influence on cross-frequency phase-amplitude coupling measures, which has been discussed extensively in the literature (Hyafil, 2017; Kramer et al., 2008; Lozano-Soldevilla et al., 2016), which may lead to incorrect inference, e.g., regarding the directionality of influence between rhythms. These spurious measures are especially a problem for the human alpha-rhythm because of its high magnitude, which could obfuscate genuine relationships, for example, in the low-beta band. As outlined above, the existence of harmonic beta-activity is common for central and posterior rhythms in the alpha-band, which means that a contamination of genuine beta-activity by harmonic beta contributions is to be expected in those regions (Furman et al., 2020; Nikulin & Brismar, 2006).

The resting state analyses presented here are relevant for any kind of baseline condition, before a stimulus was shown or a movement was performed, as well as for investigations of resting state beta-activity. Resting state recordings are a widely used recording type, especially for clinical populations. Sensitive biomarkers derived from EEG would be highly valuable, for example, to aid diagnosis or monitor treatment outcome (Başar et al., 2013; de Aguiar Neto & Garcia Rosa, 2019; Mussigmann et al., 2022). Since in this domain, analysis is often guided by canonical frequency bands, effects that are observed in the beta-frequency band could potentially stem from alpha-band effects, while other effects might remain unnoticed due to the reduced influence of genuine beta-activity. Neglecting to distinguish between different rhythm types may result in decreased specificity in approaches based on beta-band activity. Although our demonstrations are based on resting state EEG data, we acknowledge that the composition of beta-activity may differ in a task setting, with a higher prevalence of genuine beta bursts during or post movement, for instance. By carefully differentiating temporal dynamics as well as source locations, it may be possible to identify unique contributions of genuine beta-rhythms, such as in the case of postmovement beta rebound (Jurkiewicz et al., 2006), in contrast to a more global event-related desynchronization processes (Pfurtscheller & Neuper, 1994) that are often of comparable dynamics across mu- and beta-frequency bands and therefore may be of a harmonic nature.

A refined distinction between harmonics and genuine beta-rhythms could be especially beneficial for investigations of sensorimotor rhythms, where multiple rhythm types collide across a small space of cortical areas (Salmelin & Hari, 1994; Salmelin et al., 1995). Studies in rats have even reported a somatosensory beta-rhythm in addition to a motor beta-rhythm (Fransen et al., 2016). An improved understanding of waveform-related harmonic contributions vs. genuine individual cross-frequency coupled rhythms will enable a better understanding of the distinct generators of these rhythms, e.g., through computational modeling (Jones et al., 2009; Neymotin et al., 2020) or by targeting individual rhythms through real-time phase-dependent stimulation (Wischnewski et al., 2022). Therefore, it is necessary to clarify the relationship across alpha- and beta-frequency rhythms in a specific study in order to investigate functional relevance of beta-rhythms in a methodologically sound manner.

## Data Availability

The figures are generated using following available openly available data set: “Leipzig Cohort for Mind–Body–Emotion Interactions” (LEMON dataset) (Babayan et al., 2019), from which the preprocessed EEG data were used, which is available under fcon_1000.projects.nitrc.org/indi/retro/MPI_LEMON.html. Associated code to reproduce figures is available under: github.com/nschawor/eeg-beta-harmonic.
